# Preparation of Cavities for Filling Teeth

**Published:** 1855-07

**Authors:** A. S. Talbert

**Affiliations:** Lexington, Ky.


### ARTICLE XVI.

*Preparation of Cavities for Filling Teeth. Delivered before the Mississippi Valley Association of Dental Surgeons, Feb.* 23, 1855. By A. S. Talbert, D. D. S., Lexington, Ky.

Gentlemen of the Profession—By *appointment*, not *preference*, I appear before you to read a paper. While I appreciate the honor thus conferred upon me, I distrust my abilities to *entertain* or *instruct* even a *few* of the large number present; many, nay, *most* of whom are *older* than myself. I will not indulge in fairy fields of fancy; I will not elaborate on new theories, or harp upon the downfall of exploded ones, nor yet will I endeavor to please the imagination by a delightful play upon words ("without knowledge;") believing as I do that *practical* truths, however plainly clad, are better calculated to fulfill the ultimate objects of our association. Profiting by the intelligence of my audience, I need not give in detail a description of the various classes of teeth—their development, their anatomical structure, their chemical analysis, their relation one to another, and as a whole to the general system; nor need I speak of their division into front, lateral, lingual, and other surfaces ascribed by anatomists to their various parts, for with all these you are equally familiar. I propose, therefore, to arrange all cavities into two classes—those that require the use of the file, and those that are accessible without this instrument.

Since the venerable key has been laid aside, there is no instrument found in our cases that imparts so invariably on its appearance to our patients, that instinctive shudder, that indescribable shrinking from its use, as does the file. If the skillful limner would draw accurately the outlines of distorted visage, let him place his canvass in front of one of our operating chairs. Let the operator on a *cold* day, dip his cold dull file into cold water, let him commence on the sensitive teeth of a nervous patient, and as the old file begins to warm by its friction, and to cloy by its accumulation, and the temperature of his energies begins to rise in like proportion, and he hastens his hand lest he may not get another opportunity—let the artist seize his pencil, and if he draws his picture true to nature, he will, to say the least of it, have a "*wry face!*"

There is no instrument that has suffered so much abuse, or that has been the subject of so many and such ridiculous imputations. If teeth are badly filled and their fillings fall out, their loss and the consequent decay of the teeth are attributed to their having been filed; and not unfrequently sound teeth have decayed rapidly; because teeth in a distant part of the mouth had been filed by some itinerant dentist!! Who, that has not heard the remark repeatedly from his patients, "my teeth were all sound except the least speck in one of the front ones, when a certain Doctor ——, filed them and filled it—his filling come out, and since then they have *all decayed!!*" forgetting that they might have *all decayed* just the same, if she or he had never seen a dentist. As calomel gives them "*miseries*" in all their bones, so filing gives them a *misery* in all their teeth. Hard, however, as the nether millstone, it has cut its way through every obstacle in the hands of an intelligent profession, till its probang objectors have dwindled into a respectable minority, whose influence no longer deters the enlightened dentist from the commencement and completion of his operation according to the dictates of his own judgment. Having dipped a clean file into tepid water, if the front incisors above, be decayed on their lateral surfaces, the operator will stand behind, and a little to one side of his patient, commencing with a thin flat file, each cutting edge of equal thickness, and as the operation advances, he will once, twice, or thrice, as the case may require, dip again his file in the tepid water to wash away the adhering portions of the filed tooth. In like manner, thick ones will be used till the objects are attained. Care being taken to leave sufficient shoulders of healthy enamel or bone upon the necks of the teeth, to prevent their changing positions, as they might otherwise do, some *approaching*, others *receding* from each other.

By these shoulders a better shape may be retained in the teeth, than if the desired object were attained by means of a wedge-shaped file. After the file, a sharp instrument, such as is used in scaling and cleaning teeth, will be required to round the edges of the enamel and smooth their surfaces.

The objects of the file are twofold—to make *room*, and to remove such portions of enamel as may be deprived of its vitality. False pride on the part of the patient accompanied by false delicacy on the part of the operator should not deter him from attaining to both of these, for on them depends the success of all that are to follow.

The preparation of a cavity in a tooth is a fixed fact, as well as its filling. Unlike the homœopathist, we cannot give a few globules of the essence of the tenth part of the shadow of *nothing*, combined with a little sugar of milk, and *let nature* do the work for us, while we "steal her *thunder*" by playing upon the *credulity* of our patients.

Having obtained the required space and removed such portions of the enamel as may be necessary, we are prepared to *enter* the *cavity*. Three instruments only are necessary—the hatchet, the hoe and the *hard drill.* Right and left curved excavators; excavators bent upon themselves in two or three directions as we often see them, are good for nothing in removing decay, and should be discarded.

But having thus cut away the caries with the hatchet and hoe, we have found the cavity not of a suitable shape to retain the filling. We are now ready for the drill. It is simply a worn out excavator, which having served out its time in *cutting* among the "dry bones," and been laid aside as useless, is now to have its neck broken off—and having been deprived of its *temper*, it is to be somewhat reversed in shape; its point being left a very little larger, which is to be filed from both ways, meeting in the centre, forming a straight edge. When hardened as hard as fire and water will make it, then polished, it is ready for use.

With these exceedingly hard and sharp drills you have only to make a few rotations, at any point or points in the cavity that may best suit your convenience, or that the condition of the decay may demand; care being taken to hold a steady hand, especially in the larger ones, which, however, need not be larger than an ordinary knitting needle, or one-sixteenth part of an inch in diameter, while the smaller ones may be one-fourth as large or even less.

The operator, unaccustomed to the use of this instrument, will be astonished at the rapidity with which he is now able to prepare points in the cavity which shall serve as fastenings to his plug. He is also better enabled to make choice where these points shall be cut, selecting them remote from the pulp, and at such places as it would be impracticable to use an ordinary excavator.

Having finished the internal walls of the cavity with these three instruments, the hatchet, the hoe, and hard drill, it is completed with the burr by a few rotations of this instrument on the enamel, forming the edges of the cavity.

It will be remembered, we have been operating on a front tooth after the use of the file. It is asked, how will you make a straight instrument enter the cavity, so as to fulfill the indications required? It is answered. Suit your instrument to the width of space obtained by the file, taking advantage of the angle found, by placing the drill obliquely across the space, and making your points in the upper and lower, the outer and inner walls of the cavity.

If I may be allowed to digress a moment, I will smply enter protest against all separations produced by India rubber, gutta percha, wooden wedges, cotton or anything else, for granting that teeth may be thus separated *without injury* to either themselves or surrounding parts, the plug itself cannot be *finished* without the file.

Having thus finished all the front cavities, we are prepared to commence the posterior surface of the cuspidati. The same instruments are required in addition to a thick straight wedge-shaped file, by the free use of which you will obtain three important points, space, the removal of all softened bone, and clean smooth finish to your work.

As we advance toward the dens sapientiæ, the file requires to be so bent upon itself as to protect the angle of the mouth, but no other new instrument is required in the front approximal surfaces, nor is the hard drill dropped till we have the posterior surface of the first molar, when in its stead our hatchets are to be bent more at right angles. The *burr* is not to be forgotten in finishing the mouth, if I so speak of all these cavities, when it is practicable to reach them with it.

In filling the inferior teeth, no new instrument is required, hence, no description further is necessary, for the first class of cavities.

We come then to the consideration of the second class, to wit: such as are accessible without the use of the file. These are found in the grinding surfaces of the molars and bicuspids in the labial and lingual surfaces, or in such approximal surfaces as have been exposed by the extraction of the adjacent teeth.

In the preparation of these, we are to supply the file with heavy cutting instruments of various shapes, but chiefly the hatchet and chisel; with which every thin and softened fragment of enamel is cut away, following every ramification to its farther extent. Having cut away the caries with the excavator, the operator is ready for the hard drill, with which to make a suitable shape to the cavity, a few turns of it only being necessary to penetrate healthy dentine, or even enamel, as is often required in following the ramifications of incipient decay in the grinding surfaces of the molars and bicuspids. On the other hand, if these surfaces be badly decayed, they are to be cut in like proportion, even to the removal of one half the crown of the tooth, either transversely or otherwise, as the case may be.

But we have been treating such cavities as require no medication,—teeth, the nerves of which are not exposed in the preparation of their cavities.

Returning again to the superior central incisors, we have found the pulp exposed, but in a healthy condition. It is to be cut off and extracted by the use of softened steel, silver or gold instrument, which are to be rapidly inserted into the nerve cavity to the apex of the root, when with a quick turn, it is withdrawn, bringing along with it the pulp of the tooth. After washing it well with warm water, to which is added a little pure brandy or spirits camphor, and thoroughly drying, it is ready for filling. Should our patient refuse to submit to this *surgical* treatment, we are to destroy the vitality by medication. Care is to be taken not to push the treatment too far; it only being necessary to *paralyze* the nerve, and not destroy it with medicine. There is, perhaps, no better preparation with which to promote this object, than a paste of the consistence of common putty, made by rubbing well together in a mortar, three parts by weight of arsenious acid and one of acetate or sulphate of morphia, with sufficient creosote to make the paste. This may be always kept on hand in a close stopped vial. Its plasticity enables the operator to place it directly on the pulp, if properly exposed; while its solubility prevents it producing more than momentary pain by its mechanical pressure.

Being well stopped in by a little cotton or common bees-wax, the patient is less annoyed by its unpleasant taste, than if used in the form of consistent fluid, as is sometimes recommended, the operator having more time to stop it well before the fluids of the mouth have diffused it over its surface. A globule the size of a homœopathic pill, is sufficient to produce the desired result.

After allowing it to remain from a few minutes to as many hours, without much regard to time, the paralyzed nerve and vessels are cut away as before described, the blood running freely, with however little or no pain. Complete the operation as before, and it will be successful in ninety-nine cases in a hundred, depending upon the diagnosis between filling and extracting, togther with the treatment. Of the remaining teeth in which the nerves are to be destroyed previous to filling, it is hardly necessary to speak, since their treatment is to be conducted in the same way, varying a little to suit their position; becoming more difficult, with their increased number of fangs.

For the sake of preserving the natural appearance of the crowns and front surfaces of the incisors, they may cut chiefly on the labial surface, after the free use of the flat file. Should the nerve cavity still be inaccessible, the operation may be facilitated by drilling through the enamel of the lingual face, directly opposite the inferior apex of the pulp, thus forming an opening continuous with the nerve cavity directly through the tooth, by which the root may be easily washed with the syringe and the whole thoroughly filled. The advocates of risodontropy will join me in testimony to the use of the hard drills in this operation; though I suppose the profession and community would have been quite as well served if they had never been used for any other purpose, than that of preparing cavities for filling, instead of boring good for nothing holes in teeth to be left unstopped, nucleus for filth and disease. But, it is not the province of this paper to discuss the utility of risodontropy.

We have one more class of cavities to treat, and I have already passed the limits of brevity which I prescribed in my commencement; I allude to those cavities in which the nerves had been destroyed previously by inflammation or otherwise. They require a more careful diagnosis, a more patient and longer treatment preparatory to filling them. We are to look well to our patient's diathesis, and if it be scrofulous, rheumatic, or otherwise prone to disease, we shall decide in favor of the extraction of the tooth; but if the general health be good and the whole mouth be made healthy by the entire removal of fangs and irrestorable teeth, the operator will proceed to remove every part of the remains of the pulp, washing well with warm water, to which has been added an equal part of chloride of soda, a little creosote or chloroform.

Should, however, the disease have extended further, involving the apex of the root and its membranes, the treatment must be continued successively from day to day, until the effusion of purulent matter ceases to exhibit the least symptom of disease within, and the external appearance of the gum shows a healthy action. When these indications are fulfilled, it may yet be well to insert a temporary filling of any substance that will perfectly exclude the air and moisture, and stop up any exhalations evolved by the action of disease that may chance to continue at the apex of the fang.

After this temporary filling has been worn forty-eight hours without pain or inconvenience, it may be removed, and the operation completed, with a confidence that will justify the effort, even though it prove occasionally unsuccessful.

Thus I have endeavored to notice all the preliminaries, as well as the necessary manipulations preparatory to inserting fillings in teeth! More might have been said,—less might have been told with better effect.

Mine is not the pen of a ready writer, nor did I suppose till within a few days, that I should find it in my power to fill my appointment. I regret that what I have done is not more worthy the confidence of those who conferred the duty upon me. Wherein my pen has failed, I shall be happy to demonstrate on the living subject, or answer any questions you may suggest. In conclusion, I thank you for your kind attention.—*Dental Register.*

